# Parental perceptions of school safety and institutional response after a mass school shooting in Serbia

**DOI:** 10.3389/fpubh.2026.1703138

**Published:** 2026-04-07

**Authors:** Miodrag Stankovic, Aleksandra Stojanovic, Maja Simonovic, Vladimir Djordjevic, Milica Mitrovic

**Affiliations:** 1 Medical Faculty, University of Nis, Niš, Serbia; 2 Centre of Mental Health Protection, University Clinical Centre of Nis, Niš, Serbia; 3 Clinical Hospital Center “Dragisa Misovic – Dedinje”, Belgrade, Serbia; 4 Department of Psychology, Faculty of Philosophy, University of Nis, Niš, Serbia

**Keywords:** perception of security, risk factors violence prevention, school shootings, school violence, student safety

## Abstract

**Background:**

Serbia's first fatal school shooting on 3 May 2023, followed by a subsequent mass murder, resulted in widespread societal distress.

**Objective:**

This study aimed to assess the emotional, cognitive, and behavioral responses of parents of school-aged children who were neither directly nor indirectly exposed to the events. It also examined parental perceptions of children's safety at school, access to information and psychological support, attitudes toward proposed preventive measures, and trust in institutional capacity to prevent future incidents.

**Methods:**

A total of 1,996 parents and caregivers were recruited through a stratified survey-delivery method, with the Ministry of Education distributing the survey to schools, which then forwarded it to parents. The pilot study commenced 60 days after the Belgrade school shooting at “Vladislav Ribnikar” Elementary School, and the confirmatory phase began 12 months later. Parents of children directly or indirectly involved in the shooting were excluded.

**Results:**

Of the 1,927 parents who participated, over 98% reported no direct or indirect exposure to the shooting. Nevertheless, 69% expressed concern about their children's safety at school. More than half (56%) attributed responsibility to the underage perpetrator, and 65.7% supported lowering the age of criminal responsibility, most commonly to 12 years. Although 46% perceived psychological support as accessible, actual utilization was minimal (3.8% among children; 2.9% among parents). Parents predominantly favored strict enforcement of child protection laws over security or mental health–based preventive measures. Institutional trust was low, with 60.6% reporting minimal confidence in authorities' ability to prevent similar events.

**Conclusions:**

The findings indicate that Serbia's first mass school shooting had broad community-level effects, including heightened parental safety concerns, low institutional trust, and minimal use of psychological services. These results highlight the need for transparent communication, strengthened institutional trust, and integrated prevention strategies that include accessible psychosocial support.

## 1 Introduction

School shootings are typically defined as incidents in which a student intentionally shoots and injures or kills at least one peer or staff member on school grounds, excluding interpersonal disputes or gang-related violence (1). Such events are generally public, involve indiscriminate targeting, and the perpetrator's identity is not concealed (2).

On May 3, 2023, Serbia experienced its first mass school shooting when a 13-year-old student killed nine peers and one staff member and injured several others at “Vladislav Ribnikar” Elementary School in Belgrade. The following day, a separate shooting occurred in the villages of Dubona and Malo Orasje, resulting in additional casualties; however, because it did not occur inside a school and did not involve a student perpetrator, it did not meet the definition of a school shooting.

These incidents prompted a nationwide psychological, social, and political response, including public protests, increased media coverage, emergency firearm-reduction policies, and the deployment of police officers to urban schools.

While extensive research has examined children's and communities' reactions to mass violence, particularly in the United States, limited knowledge exists regarding parental responses in countries without prior experience of school shootings. For instance, after the Uvalde school shooting in 2022, 32% of 3,757 surveyed U.S. parents reported being very or extremely worried about a shooting at their child's school (3). Research following the Columbine school shooting demonstrated that such events generate widespread emotional, cognitive, and behavioral reactions not only among directly exposed families but also within the broader community (4).

Immediate post-event interventions focus on restoring a sense of safety, ensuring access to mental health services, and restricting children's access to firearms, especially during crises or when mental health symptoms are present (5–9). However, there remains a gap in the literature regarding parental responses in contexts without prior exposure to school shootings.

Despite extensive global research on school shootings, no prior study has examined parental perceptions, emotional responses, and perceived safety following Serbia's first mass school shooting or within a sociocultural context lacking previous exposure to this form of violence.

This study aimed to assess the emotional, cognitive, and behavioral responses of parents of school-aged children who were neither directly nor indirectly exposed to the events. Specifically, it examined parental perceptions of children's safety at school after the incident, access to information and psychological support, attitudes toward proposed preventive measures, and trust in institutional capacity to prevent future incidents.

Understanding parental responses in this context is essential for informing future mental health interventions, shaping school safety policy, and strengthening institutional crisis response systems.

## 2 Subjects and methods

### 2.1 Study design

A two-phase, cross-sectional survey design was employed to assess parental emotional, cognitive, and behavioral responses following the first mass school shooting in Serbia.

Phase 1 was conducted 60 days after the event (local pilot survey), while Phase 2 was implemented 12 months later as a nationwide survey.

### 2.2 Sampling and recruitment

#### 2.2.1 Phase 1 (pilot study)

Participants were recruited using convenience and snowball sampling. Survey links were disseminated via email, social networks, and direct communication with school psychologists and directors, who then forwarded the links to parents.

#### 2.2.2 Phase 2 (nationwide study)

In Phase 2, the Ministry of Education distributed the survey through its internal communication platform to all primary and secondary schools. Schools were instructed to inform parents about the study and invite voluntary participation. The number of parents who received the invitation is unknown; therefore, the response rate could not be calculated.

### 2.3 Participants

Parents or primary caregivers of school-aged children (elementary and secondary schools) were eligible to participate. Exclusion criteria included: direct or indirect exposure to the school shooting; inability to provide informed consent; and lack of Serbian language proficiency.

A total of 1,996 parents and caregivers participated (86% mothers, 12% fathers, 2% other relatives or foster parents).

Direct and indirect exposure to the school shooting events was assessed using two self-report screening questions. Direct exposure was defined as the respondent or a member of their immediate or extended family being physically present at the attack site during the events. Indirect exposure was defined as a contextual or relational connection to the event without physical presence during the attacks, such as having a child enrolled in the affected school but not present at the time of the incident (e.g., attending a different shift). Participants were asked whether they or any family member had been directly exposed to the attacks, and separately whether they or any family member had been indirectly exposed. Respondents who reported either direct or indirect exposure were excluded from the analytic sample in order to focus the analysis on parents without personal or familial exposure to the events.

### 2.4 Survey instrument

The survey comprised 50 items assessing emotional reactions, perceived safety, access to support, attitudes toward preventive measures, and institutional trust.

Survey questions assessing parental attitudes toward school shootings were developed for the present study. The questionnaire design was informed by the general survey structure and response formats of a Caregiver Needs Survey developed by Global Autism Public Health Initiative for Autism Speaks, previously used by the author during the COVID pandemic (28), although all items were newly written and tailored to the context of school shootings.

The questionnaire covered nine domains, including demographic characteristics; exposure to the event; direct and indirect impact of the event; impact of information and media exposure; continuity of support and care services; parental and family functioning following the event; barriers to and facilitators of recovery and adaptation; development of psychological symptoms and disorders in children and parents; and risk mitigation and prevention of future adverse outcomes.

### 2.5 Procedure

Phase 1 data collection occurred during July 2023 (60 days after the event). Phase 2 data collection occurred 12 months after the shooting, from May to the end of November 2024. Parents accessed the survey via an anonymous Google Forms link. Written electronic informed consent was obtained before participation.

The pilot form questionnaire contained 50 questions in the Serbian language, designed to be completed within 15–20 min by online link:

- https://docs.google.com/forms/d/e/1FAIpQLSejStlDOPEv9egDM21zNjXpKXBc5G_l_uQPua8e_9o_uJzDXQ/viewform?usp=sf_link

The final form did not contain questions that were not approved by the Ministry of Education, but it had replacement questions, and in the end, it also had 50 questions in the Serbian language and can be found at the link:

- https://docs.google.com/forms/d/e/1FAIpQLScbWK13o5aeCjW4tIZDFItsgAvF7rZTmtKZuk8HjO42fVUQ/viewform?usp=sf_link

The survey was intended for parents or primary caregivers of all school children from elementary and secondary schools. In the first phase, it was local regia, and in the second, it was from all parts of the country.

Following review by the Ministry of Education, some items were removed due to concerns related to public sensitivity. Removed items included questions regarding the use of lethal force by school police officers. One such question was: “Is a school police officer allowed to shoot an armed child who refuses to surrender and poses a threat?” Replacement items were added to maintain thematic coverage while adhering to Ministry guidelines. Also, the Ministry suggested that the questionnaire should not be given to children at all until the Republic Expert Commission approves it.

### 2.6 Data analysis and statistical analysis

Although the questionnaire was structured around nine predefined domains, the results were organized into broader thematic groups. These categories were developed during data analysis by integrating conceptually related findings across multiple domains to enhance interpretability and coherence.

Participants reporting direct or indirect exposure to the events were excluded from inferential analyses. Group differences between mothers and fathers were examined using independent-samples *t*-tests for continuous variables, and Mann–Whitney *U*-tests for ordinal variables. Categorical variables were compared using chi-square tests. Effect sizes are reported as Cohen's *d* and Cramer's *V*, and 95% confidence intervals were calculated. Composite scores were calculated as the mean of constituent items, where conceptually appropriate.

### 2.7 Ethics

The study was approved by the Ethical Committee of the University Clinical Center Nis (No. 19486/11, 7 July 2023). All participants were informed about the study, and all provided informed consent via an online system.

## 3 Results

For clarity, results are presented in thematic sections that do not correspond directly to the original questionnaire domains. Thematically related items from different domains were grouped to reflect higher-order patterns in parental perceptions and responses (Table 1).

**Table 1:** Mapping of survey domains to thematic result group.

Thematic result group (results)	Corresponding questionnaire domains (instrument design)
Sample characteristics and demographics	Demographic characteristics
Perceptions of perpetrator responsibility	Exposure to the event; Direct and indirect impact of the event; Information and media exposure
Information availability and expert guidance	Impact of information and media exposure; Continuity of support and care services
Access to psychological support and media information	Continuity of support and care services; Barriers to and facilitators of recovery and adaptation
Safety perceptions	Direct and indirect impact of the event; Parental and family functioning after the event; Risk mitigation and prevention
Communication with government representatives	Impact of information and media exposure; Continuity of support and care services; Barriers to and facilitators of recovery and adaptation
Short-term and long-term preventive measures perceived by parents	Risk mitigation and prevention; Preparedness-related items within exposure and impact domains
Trust in institutional capacity	Continuity of support and care services; Preparedness; Barriers to and facilitators of recovery and adaptation

### 3.1 Sample characteristic and demographics

More than 98% of respondents and their children were not directly or indirectly exposed to mass violence, indicating a high degree of sample homogeneity. A total of 1,996 parents and caregivers completed the questionnaire, with 42 participants excluded for direct or indirect exposure. The final sample of 1,927 parents (86.4% mothers, 12.4% fathers, and 1.2% other relatives or foster parents) allows for the estimation of attitudes with an approximate margin of error of ±2.19 percentage points at the 95% confidence level.

The average age of the parents at the time of the survey was 42.6 years (SD 4.5; range: 24–62). The majority of participants lived in urban areas (58% *n* = 1,158), followed by rural areas (27.5%, *n* = 549) and suburban settlements (14.5%, *n* = 289). The largest number of parents completed high school (43% *n* = 858), 28% (*n* = 559) had a college or a university degree, 18% (*n* = 359) had some part of university education, 8% (*n* = 160) had a master's degree, and 1.5% (*n* = 30) had a doctoral degree. A total of 15% (*n* = 299) of parents are single, while 2% (*n* = 40) of children lived in households in different configurations (mother/stepfather, mother/grandfather, foster families, grandmother/grandfather). The average age of the children of the parents who filled out the questionnaire was 13, and the gender distribution was equal (50.2% female and 49.8% male). Nearly all children (99%) were enrolled in public primary or secondary schools (Table 2). Over 90% of respondents identified as Serbian nationals.

**Table 2:** Sample characteristics.

Variable	Mothers (*n* = 1,665)	Fathers (*n* = 238)	Total sample	Test	*p*-Value	Effect size
Parent age (years)	42.02 ± 6.01	45.81 ± 6.32	42.6 ± 4.5	*t*-test	<0.001	*d* = −0.63
Child age (years)	12.96 ± 3.36	13.31 ± 3.33	13.0 ± —	*t*-test	0.138	*d* = −0.10
Parent sex (%)	86.4%	12.4%	—	—	—	—
Child sex (% female)	51.3%	49.6%	50.2%	χ^2^	0.505	*V* = 0.02
Education (higher education)	62.8%	60.9%	—	χ^2^	0.487	*V* = 0.05
Employment (employed)	71.4%	84.0%	—	χ^2^	<0.001	*V* = 0.17
Place of residence						
Urban	—	—	58.0% (*n* = 1,158)	—	—	—
Rural	—	—	27.5% (*n* = 549)	—	—	—
Suburban	—	—	14.5% (*n* = 289)	—	—	—
Marital status (single parent)	—	—	15.0% (*n* = 299)	—	—	—
Other household configurations^*^	—	—	2.0% (*n* = 40)	—	—	—
Children enrolled in public schools	—	—	99.0%	—	—	—
Nationality (Serbian)	—	—	>90%	—	—	—

Values are presented as mean ± standard deviation or percentage. The analytic sample consisted of 1,927 parents, after excluding respondents who reported direct or indirect exposure to mass violence.

*Other household configurations include mother/stepfather, mother/grandfather, foster families, and grandparent-led households.

### 3.2 Perceptions of perpetrator responsibility

A total of 56% (*n* = 1,018) of parents who answered, consider the underage perpetrator responsible for the school shooting, while 28.6% (*n* = 519) described him as a “monster” (reflecting prevalent media framing). Another 10.5% (*n* = 190) were undecided, 4.3.9% (*n* = 78) believed a child could never be guilty, and only 0.7% (*n* = 12) considered the child not responsible [χ^2^_(4)_ = 1,043.60, *p* < 0.001]. A total of 110 parents did not answer this question.

In response to the question: “In your opinion, at what age should a child become criminally responsible?”, the majority of parents expressed the view that the age of criminal responsibility should be lowered below 14 years (65.1%; *n* = 1,092). Most frequently, parents identified the age of 12 years as the appropriate threshold (42.9%; *n* = 720; Table 3). A proportion of parents (12.9%; *n* = 249) indicated that they either did not know or did not consider themselves competent to provide an answer to this question. The distribution of preferred ages of criminal responsibility differed significantly from an equal distribution across categories [χ^2^_(5)_ = 1,078.62, *p* < 0.001; filtered sample, valid responses only]. Differences between mothers and fathers were examined using the Mann–Whitney *U*-test. Fathers tended to endorse a significantly higher age threshold for criminal responsibility than mothers (*U* = 127,431, *z* = −4.50, *p* < 0.001), with a small but statistically reliable effect size (*r* = 0.11).

**Table 3:** Preferred age of criminal responsibility (aggregated categories; filtered sample; question: “In your opinion, at what age should a child become criminally responsible?”).

Age category (years)	Mothers *n* (%)	Fathers *n* (%)	Total *n* (%)
6–9	39 (2.5)	4 (2.7)	43 (2.5)
10–11	305 (19.5)	24 (16.0)	329 (19.3)
12	675 (43.1)	45 (30.0)	720 (42.3)
13–14	276 (17.6)	29 (19.3)	305 (17.9)
15–16	190 (12.1)	21 (14.0)	211 (12.4)
17–18+	81 (5.2)	27 (18.0)	108 (6.3)
Total valid responses	1,566 (100.0)	150 (100.0)	1,716 (100.0)
Missing/non-codable	99	88	187
Total (filtered sample)	1,665	238	1,903^*^

Differences between mothers and fathers in the preferred age of criminal responsibility were examined using the Mann–Whitney U-test on valid numeric responses only. Fathers tended to endorse a higher age threshold than mothers (U = 127,431, z = −4.50, p <0.001), with a small but statistically reliable effect size (r = 0.11). Respondents who selected “I do not know” or provided non-numeric answers were excluded from this analysis. ^*^Filtered sample after exclusion of missing and non-codable responses.

### 3.3 Information availability and expert guidance

A total of 39% (*n* = 778) of parents felt adequately informed about the event, while 12.3% (*n* = 246) felt insufficiently informed (χ^2^_(1)_ = 276.39, *p* < 0.001). Regarding expert commentary, 13.2% (*n* = 263) reported that it was excessive, 15.2% (*n* = 303) found it sufficient, 21% (*n* = 419) perceived it as insufficient, and 15% (*n* = 299) reported receiving none [χ^2^_(3)_ = 42.92, *p* < 0.001]. Additionally, 35% (*n* = 699) reported that expert input did not help them understand how such events could be prevented, while 16.5% (*n* = 329) believed it did [χ^2^_(1)_ = 133.17, *p* < 0.001].

### 3.4 Access to psychological support and media information

When asked, “How easy was it to secure psychological support for your family?”, 47.5% (*n* = 916) of parents responded that it was “easy” or “very easy,” while 19.8% (*n* = 382) reported that it was “difficult” or “almost impossible.” The overall distribution of responses across all answer categories differed significantly [χ^2^_(4)_ = 518.62, *p* < 0.001]. The question assessed perceived availability of support rather than whether such support had actually been sought or needed. Regarding access to accurate media information about school safety, 35.2% (*n* = 679) of parents reported that obtaining such information was easy, whereas 34.2% (*n* = 659) reported difficulty. Response distribution across categories was also statistically significant [χ^2^_(4)_ = 231.39, *p* < 0.001]. Similarly, 34.7% (*n* = 669) of parents found it easy to obtain media guidance on how to communicate with emotionally distressed children, while 32.1% (*n* = 619) reported difficulty obtaining such guidance. Again, responses differed significantly across categories [χ^2^_(4)_ = 340.47, *p* < 0.001]. Helping their child express emotions related to the incident was rated as easy or very easy by 49.0% (*n* = 945) of parents, whereas 16.2% (*n* = 313) found this difficult or extremely difficult, with responses showing significant variation across categories [χ^2^_(4)_ = 610.13, *p* < 0.001]. Finally, when asked whether psychological or psychiatric help would have been accessible had it been needed, 39.7% (*n* = 764) of parents reported that such help would not have been available, either by phone or video consultation, while 21.1% (*n* = 407) believed that it would have been available. Distribution across response categories was again statistically significant [χ^2^_(2)_ = 130.71, *p* < 0.001]. Detailed item-level results are provided in Table 4.

**Table 4:** Parental perception of psychological support and information availability.

Item assessed	Easy/available *n* (%)	Difficult/unavailable *n* (%)	χ^2^ (df)	*p*-Value
Securing psychological support for the family	916 (47.5%)	382 (19.8%)	518.62 (4)	<0.001
Obtaining media information on school safety	679 (35.2%)	659 (34.2%)	231.39 (4)	<0.001
Obtaining media guidance on communication with distressed children	669 (34.7%)	619 (32.1%)	340.47 (4)	<0.001
Helping child express emotions after the incident	945 (49.0%)	313 (16.2%)	610.13 (4)	<0.001
Availability of psychological/psychiatric help if needed^*^	407 (21.1%) available	764 (39.7%) unavailable	130.71 (2)	<0.001

^*^Variable was analyzed as a categorical (available vs. unavailable) measure, which is reflected in the different degrees of freedom reported for this item.

Only a small proportion of respondents reported seeking professional mental health support following the tragedy. Specifically, 3.8% (*n* = 73) reported seeking professional help for their child, while 2.9% (*n* = 55) reported seeking such support for themselves. There was no statistically significant difference between mothers and fathers in seeking professional help for their child [χ^2^_(1)_ = 1.72, *p* = 0.190], but mothers reported having sought professional mental health support for themselves statistically significantly more [χ^2^_(1)_ = 6.96, *p* = 0.008; Table 5].

**Table 5:** Seeking professional mental health support for children and parents, by parental role.

Type of support	Mothers: yes *n* (%)	Mothers: no *n* (%)	Fathers: yes *n* (%)	Fathers: no *n* (%)	Total yes *n* (%)	Total *N*
Child sought professional help	68 (4.1)	1,597 (95.9)	5 (2.1)	233 (97.9)	73 (3.8)	1,927
Parent sought professional help	55 (3.3)	1,610 (96.7)	0 (0.0)	238 (100.0)	55 (2.9)	1,927

Child sought professional help: χ^2^_(1)_ = 1.72, p = 0.190.

Parent sought professional help: χ^2^_(1)_ = 6.96, p = 0.008.

### 3.5 Safety perceptions

Following the mass shooting, 37% (*n* = 739) of parents believed that children should return to school, whereas an equal percentage believed that they should not. A total of 39% (*n* = 778) agreed that suspending the school year was an appropriate response by the education system, while 27% (*n* = 539) disagreed [χ^2^_(1)_ = 43.37, *p* < 0.001].

Regarding perceived safety for whole sample divided in categories concern/no concern, 69% (*n* = 1,377) of participants expressed concern for their children's safety at school, while 14.6% (*n* = 289) reported no concern [χ^2^_(1)_ = 710.53, *p* < 0.001]. Mothers reported significantly higher levels of worry across all safety-related indicators compared with fathers, including concern for their own safety, their child's safety, and fear of losing a loved one (all ps <0.001; small-to-moderate effect sizes). The composite Safety/Worry score was substantially higher among mothers than fathers (*d* = 0.58; Table 5). Fathers expressed greater confidence in their own and their child's ability to manage safety-related situations, while no significant differences were observed in confidence that the state could prevent similar events. Mothers reported greater difficulty refraining from repeated monitoring of event-related information than fathers (*d* = 0.52). No group differences were observed in perceived sufficiency of information or expert explanations. Mothers more frequently reported seeking professional psychological help for themselves following the events, whereas no group differences were observed in help-seeking for children (Table 6).

**Table 6:** Differences between mothers and fathers in perceived safety, concern, and institutional trust.

Item (abbreviated)	Mothers *n*	Mothers M (SD)	Fathers *n*	Fathers M (SD)	*p*-Value	Effect size (*d*)
Concern for personal safety	1,665	3.01 (1.44)	238	2.40 (1.38)	1.00e-09	0.43
Concern for child's safety	1,665	4.05 (1.22)	238	3.42 (1.41)	3.72e-12	0.50
Concern about losing a loved one	1,665	3.87 (1.30)	238	3.15 (1.41)	2.58e-14	0.55
Concern about becoming a victim of a mass killing	1,665	3.09 (1.45)	238	2.28 (1.37)	1.36e-15	0.56
Ease of securing psychological support for the family	1,665	2.54 (1.27)	238	2.28 (1.15)	0.00541	0.21
Ease of obtaining useful information on school safety	1,665	2.94 (1.32)	238	2.92 (1.37)	0.915	0.01
Confidence that the state can prevent recurrence of such events	1,665	1.78 (1.15)	238	1.90 (1.23)	0.167	−0.10
Confidence in the child's ability to cope with safety issues	1,665	3.15 (1.09)	238	3.42 (1.00)	0.00098	−0.25
Ability to refrain from constant info checking	1,665	3.34 (1.23)	238	2.70 (1.26)	0.001	0.52
Children should return to school?	1,665	2.81 (1.12)	238	3.05 (1.09)	0.002	−0.22

Group differences between mothers and fathers were examined using the Mann–Whitney U-test (two-tailed). Values are presented as means (M) and standard deviations (SD) for descriptive purposes. Effect sizes are reported as Cohen's d (positive values indicate higher scores among mothers; negative values indicate higher scores among fathers). p-Values correspond to Mann–Whitney U-tests.

### 3.6 Communication with government representatives

The majority of respondents reported no opportunity to speak with government officials responsible for school safety following the events (75.3%, *n* = 1,451), while only a small proportion reported having such an opportunity (5.6%, *n* = 108; χ^2^_(1)_ = 1,000, *p* < 0.001). The remaining respondents provided undecided or missing responses (19.1%, *n* = 368). There is no statistical difference between genders 93.5% of mothers and 90.2% of fathers reported no opportunity to speak with officials.

### 3.7 Shor-term and long-term preventive measures perceived by parents

**Figure 1:**
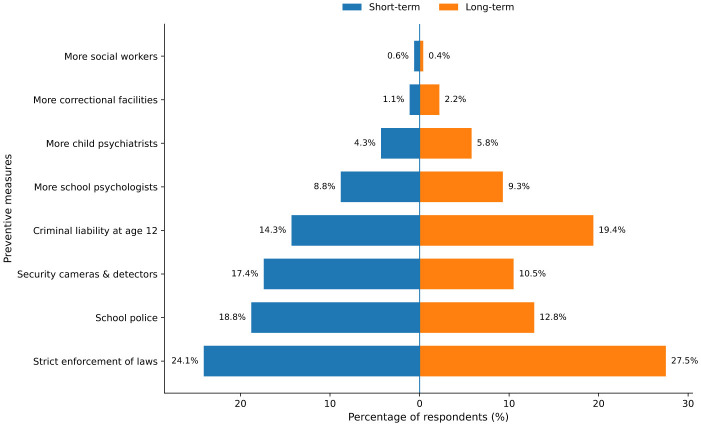
Short term and long-term preventive measures. Mothers and fathers differed modestly in preferred short-term preventive measures, whereas long-term preventive preferences were similar.

Parents' preferences regarding both short- and long-term strategies to prevent school violence were highly uneven (Table 7; Figure 1). For short-term measures, strict enforcement of child protection laws was most frequently endorsed (24.1%), followed by school police officers and technical security measures. The distribution differed significantly from a uniform distribution χ^2^_(7)_ = 706.3, *p* < 0.001. A similar but more pronounced pattern was observed for long-term measures, with enforcement of child protection laws and prison sentences for minors emerging as dominant priorities [χ^2^_(8)_ = 932.8, *p* < 0.001]. Comparison of short- and long-term priorities revealed significant shifts in emphasis [χ^2^_(7)_ = 118.5, *p* < 0.001], indicating greater long-term support for legal and punitive approaches and relatively reduced emphasis on immediate security measures.

**Table 7:** Comparison of parents preferred short-term and long-term measures to prevent school violence.

Measure	Short-term *n*	Short-term %	Long-term *n*	Long-term %
Strict enforcement of child protection laws	481	24.1	549	27.5
Prison sentences for offenders aged 12+	285	14.3	387	19.4
School police officers	375	18.8	255	12.8
Security cameras and metal detectors	347	17.4	210	10.5
Increased number of school psychologists	176	8.8	186	9.3
Increased number of child psychiatrists	86	4.3	116	5.8
Expansion of correctional facilities	22	1.1	44	2.2
Increased number of social workers	12	0.6	8	0.4
Increased number of pedagogues	—	—	14	0.7
Total valid responses	1,784	100.0	1,769	100.0
Missing/no response	143	—	158	—
Total sample	1,927	—	1,927	—

### 3.8 Trust in institutional capacity

When asked about confidence that responsible state authorities could prevent similar events, 60.6% of parents reported the lowest confidence category (*n* = 1,194), whereas 4.7% reported the highest confidence category (*n* = 92). The full distribution across the five response categories was: 1 (*n* = 1,194), 2 (*n* = 277), 3 (*n* = 302), 4 (*n* = 104), and 5 (*n* = 92; χ^2^ goodness-of-fit: χ^2^_(4)_ = 2,126.60, *p* < 0.001).

## 4 Discussion

Study provides the first systematic examination of parental emotional, cognitive, and behavioral responses following Serbia's first mass school shooting. Although participants were neither directly nor indirectly exposed to the events, the findings reveal pronounced perceptions of threat, low institutional trust, limited utilization of psychological support services, and distinct preferences for legal and punitive preventive measures. These results are consistent with prior research demonstrating that the psychological impact of school shootings extends beyond directly affected individuals and may influence entire communities through heightened vigilance, perceived insecurity, and concern for children's safety (2, 4, 10, 11).

### 4.1 Perceptions of responsibility and criminal liability of minors

More than half of parents attributed responsibility to the 13-year-old perpetrator, despite his being below the legal threshold for criminal responsibility. This divergence between legal standards and moral judgment has been described in prior analyses of public responses to juvenile violence, where responsibility is often framed in moral or symbolic terms rather than developmental or legal ones (4, 11, 12). Cross-national research on mass violence suggests that such reactions are common in societies experiencing sudden, highly publicized attacks and are often accompanied by increased public support for punitive or deterrence-oriented responses (8, 10, 13). The strong endorsement of lowering the age of criminal responsibility observed in this study may therefore reflect broader processes of social attribution and threat response rather than stable attitudes toward juvenile justice policy (11, 14).

### 4.2 Information access and expert communication

Parents reported heterogeneous experiences regarding access to information and expert guidance after the shooting. While many felt adequately informed about the basic facts of the event, a substantial proportion perceived expert commentary as insufficient or unhelpful in understanding prevention strategies. Prior studies after Columbine and subsequent U.S. school shootings have emphasized that expert-led communication plays a critical role in reducing uncertainty and supporting adaptive coping, particularly when media coverage is intensive and emotionally charged (4, 5). At the same time, studies of media framing indicate that sensationalist or repetitive reporting may amplify perceived threat and contribute to imitation effects, complicating parental interpretation of risk (15–17). The present findings suggest that although information was widely available, the lack of coordinated expert interpretation may have limited its usefulness for parents seeking guidance on prevention and family communication.

### 4.3 Access to psychological support and parental coping

Although nearly half of the respondents perceived psychological support as accessible, actual utilization of mental health services was very low for both children and parents. This discrepancy between perceived availability and help-seeking behavior has been repeatedly documented following school shootings and other mass trauma events (5, 6, 18). Research suggests that stigma, limited recognition of trauma-related symptoms, and beliefs that emotional distress represents a normative reaction may reduce engagement with mental health services, even in systems with established support structures (18, 19). Turunen et al. point out that people feel vulnerable after tragedies and have difficulty re-establishing routine, which requires long-term support (5). In our sample, almost a quarter of parents reported difficulty engaging in enjoyable activities or maintaining daily routines, while more than 40% reported restlessness and compulsive information checking, which could be signs of significant disruption from usual routines. Similar patterns of underutilization have been observed across European and North American contexts, highlighting the need for proactive outreach and normalization of psychological support following mass violence (5, 6).

### 4.4 Safety perceptions and school attendance

Parents expressed substantial ambivalence regarding their children's return to school and high levels of concern about safety within educational settings. Persistent fear of recurrence has been documented after school shootings in multiple countries, including the United States and Finland, even among families not directly affected by the incident (5, 20). Research on threat perception indicates that such concerns are shaped not only by objective risk but also by trust in institutions, media narratives, and broader societal norms regarding safety and surveillance (21, 22). These findings underscore the importance of structured reintegration strategies and school-based psychosocial interventions that address parental concerns while supporting the restoration of daily routines (29).

### 4.5 Preventive measures: legal, security, and mental health approaches

Parents in this study prioritized strict enforcement of child protection laws over physical security measures or mental health interventions. Only a minority of Serbian parents in our sample identified police officers or metal detectors as a priority. This pattern contrasts with findings from the United States, where parents more frequently endorse visible security measures such as armed officers, surveillance technologies, or metal detectors (21). Although the number of school police officers in the United States has significantly increased (65% of schools in 2019 vs. 42.8% in 2009), there is no strong evidence that their presence alone prevents school shootings (4). Countries like New Zealand and Australia have successfully implemented rigorous firearm control policies in recent decades, which has reduced the incidence of armed incidents in schools, as observed following the Port Arthur massacre and Christchurch (23). Finally, Switzerland is often cited as a high-gun-ownership country, with very low homicide rates and practically no school shooting or mass shootings (24, 25). Comparative research suggests that preferences for preventive strategies are shaped by cultural norms, perceived prevalence of violence, and attitudes toward surveillance and authority (14, 22, 23). In contexts characterized by lower baseline levels of violence and stronger social cohesion, intrusive security measures may be viewed as excessive or inconsistent with educational values (21, 23, 26).

The relatively limited emphasis on school-based mental health resources in the present sample differs from trends observed in North America and parts of Northern Europe, where mental health interventions are increasingly framed as essential components of comprehensive violence prevention (18, 27). These divergences highlight the importance of cultural context in shaping public attitudes toward prevention (30).

### 4.6 Institutional trust and crisis response

Low levels of institutional trust emerged as one of the most salient findings of this study. A majority of parents reported minimal confidence in state authorities' ability to prevent similar incidents, and most indicated they had no opportunity to communicate with responsible officials. Reduced institutional trust following mass violence has been documented in several settings, including Norway after the Utøya attack, where perceived institutional failures were associated with prolonged distress and reduced confidence among parents and youth (20). Broader research on trust and governance suggests that transparent communication, inclusive decision-making, and visible accountability are critical for restoring public confidence after crises (20, 22). The absence of such mechanisms may reinforce perceptions of institutional distance and inefficacy.

These findings underscore the necessity for transparent crisis response strategies and enhanced communication channels among educational institutions, health systems, and families.

### 4.7 Integrative interpretation

Taken together, the findings indicate that Serbia's first mass school shooting had wide-ranging psychological and social consequences extending beyond directly affected individuals. While several responses align with international patterns, such as heightened safety concerns, ambivalence about school attendance, and low engagement with mental health services, the strong emphasis on legal accountability and the pronounced lack of institutional trust reflect contextual specificities. Consistent with cross-national research on school violence and prevention, these results highlight the need for integrated approaches that combine legal enforcement, culturally sensitive communication, and accessible psychosocial support, while carefully balancing security measures with educational values and civil liberties (13, 14, 18, 21–24).

### 4.8 Limitations of study

The present study has several methodological limitations that should be considered when interpreting the findings. This sample size provides high precision for estimating proportions, but there is a gender imbalance between parents (the predominance of female respondents limits generalizability). Mothers comprised approximately 34%, with an estimated margin of error of ±2.4 percentage points; fathers comprised approximately 14%, with an estimated margin of error of ±5.9 percentage points; and foster parents comprised approximately 15%, with an estimated margin of error of ±15.5 percentage points. Accordingly, results for foster parents should be interpreted as exploratory. Other limitations: (a) The survey included study-specific items, limiting comparability to other research; (b) two-phase design limitations (differences between the pilot and final questionnaires prevent direct comparative analysis across phases); (c) self-report bias (responses may have been influenced by emotional reactions or media exposure rather than stable attitudes); (d) exclusion of directly exposed families (although ethically appropriate, this limits insight into the most affected population).

## 5 Conclusions

The findings demonstrate that Serbia's first mass school shooting generated substantial emotional and cognitive responses among parents who were neither directly nor indirectly exposed to the event. High levels of fear regarding children's safety, ambivalence about returning to school, and marked distrust in institutional capacity indicate that the psychological consequences of such incidents extend broadly across the community.

Parents prioritized strict enforcement of child protection laws as both short-term and long-term preventive measures, while comparatively fewer endorsed mental health resources or school-based psychosocial support. This pattern distinguishes the Serbian context from findings in other countries and suggests that expectations of institutional accountability strongly shape parental attitudes following mass violence.

The study further highlights the need for improved crisis communication, clearer dissemination of expert guidance, and enhanced coordination among schools, mental health services, and governmental institutions.

Overall, the results underscore the importance of strengthening preventive frameworks within the educational system, improving access to mental health support, and developing transparent, community-oriented strategies to rebuild institutional trust. Future research should explore predictors of parental safety perceptions, evaluate the effectiveness of implemented interventions, and include directly exposed families when ethically feasible to obtain a more comprehensive understanding of societal responses to school shootings.

## Data Availability

The raw data supporting the conclusions of this article will be made available by the authors, without undue reservation.
